# Fungal Colonization by *Malassezia globosa* Promotes Breast Cancer Progression and M2 Macrophage Polarization Through the MBL‐C3a–C3aR Signaling Pathway

**DOI:** 10.1002/mbo3.70193

**Published:** 2025-12-11

**Authors:** Chongwu He, Jing Chen, Ruibo Tian, Xiaoqiang Zeng, Qinyuan Han, Changan Jiang, Jun Zou, Tenghua Yu

**Affiliations:** ^1^ Department of Breast Surgery, Jiangxi Cancer Hospital the Second Affiliated Hospital of Nanchang Medical College Nanchang Jiangxi Province China; ^2^ Department of Nursing Nanchang Medical College Nanchang Jiangxi Province China; ^3^ Department of Thyroid and Breast Surgery, Xing'an League People's Hospital Xing'an League Ulanhot Inner Mongolia Autonomous Region China; ^4^ Jiangxi Medical College Nanchang University Nanchang Jiangxi Province China; ^5^ Department of General Surgery Poyang County People's Hospital Shangrao Jiangxi Province China; ^6^ Department of Abdominal Oncology Surgery Jiangxi Cancer Hospital Nanchang Jiangxi Province China; ^7^ Department of Breast Surgery, Jiangxi Cancer Hospital, the Second Affiliated Hospital of Nanchang Medical College, Jiangxi Clinical Research Center for Cancer JXHC Key Laboratory of Tumor Microenvironment and Immunoregulation Nanchang Jiangxi Province China

**Keywords:** breast cancer, C3a, fungi, macrophage, MBL

## Abstract

Fungal colonization is a known carcinogenic accomplice in lung and colon cancer but has not been implicated in breast cancer. Here, we attempt to explore the mechanism behind fungal colonization and carcinogenesis by *Malassezia globosa* in breast cancer. To begin with, we found an increased abundance of the fungus in tumor tissues of breast cancer patients and the fungal inhibitor Amphotericin‐B impeded tumor growth in patient‐derived breast cancer xenograft models. On the other hand, *Malassezia globosa* enhanced the proliferative, migratory, and invasive abilities of breast cancer cells, and facilitated tumor growth in vivo. The positive effect of *Malassezia globosa* on tumor growth occurred via M2 macrophage polarization resulting in the activation of the pro‐inflammatory MBL‐C3a‐C3aR signaling cascade which was reversed with the knockout of MBL expression. The proliferative, migratory, and invasive capacities of breast cancer cells were enhanced by culture medium from *Malassezia globosa*‐treated THP‐1 cells, which were rescued by a C3aR antagonist. In conclusion, *Malassezia globosa* activates MBL‐C3a‐C3aR signaling to trigger M2 macrophage polarization, promoting breast cancer progression and this study unravels a novel paradigm for breast cancer treatment.

## Graphical abstract

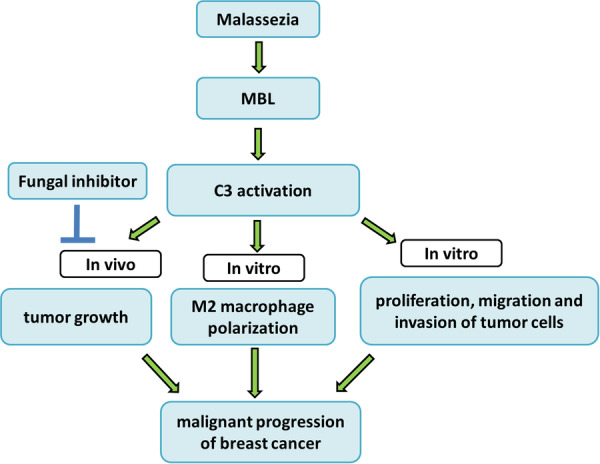

## 1 Introduction

Breast cancer is the most prevalent malignancy in women with an increasing incidence worldwide (Katsura et al. 2022; Motamedolshariati et al. 2014). According to the World Health Organization (WHO), the number of newly diagnosed cases were 2.26 million in 2020 thereby surpassing lung cancer to become the largest type of cancer in the world (Pizzato et al. 2022; Sung et al. 2021). Despite immense progress in treatment strategies including radiotherapy, surgery, adjuvant and neoadjuvant therapy, 30% of early stage patients still progress to the advanced stage and the 5‐year survival rate in advanced patients is a dismal 29% (Fisusi and Akala 2019; Weigelt et al. 2005). The pathological mechanisms regulating breast cancer progression still remain largely unknown, and further research is needed in this arena for the development of novel targeted treatment options.

Intriguingly, in recent years, the role of the host microbiome has been identified as a crucial component in the development and progression of several types of cancer including breast cancer (Huang and Mao 2022; J. Li et al. 2024). It is well known that fungi coexist with bacteria in the gastrointestinal tract, skin epithelium, respiratory tract, and reproductive organs of mammals, forming complex ecosystems of host microbe interactions (Dart 2019; Skalski et al. 2018). By promoting Th2 differentiation, commensal fungi can inadvertently facilitate cancer progression by promoting an suppressive immune microenvironment (DeNardo et al. 2009; Galloway‐Peña et al. 2024; Shiao et al. 2015). For example, supplementation with specific fungi such as *Malassezia*, *Candida*, or *Saccharomyces* exacerbates colitis (Chiaro et al. 2017; Iliev et al. 2012; Limon et al. 2019). In breast cancer, there is a negative correlation between patient survival and decreased expression of the innate immune receptor that senses fungi (Dectin‐1) (Shiao et al. 2021) and a study on the different types of fungi in breast cancer found that *Malassezia* were more abundant in breast cancer as compared to other tumor types (Saftien et al. 2023). However, the specific relevance of Malassezia in regulating breast cancer progression is yet unknown.

The complement system is a crucial component of innate immunity (Kemper et al. 2023) consisting of more than 50 soluble and membrane bound proteins, including complement intrinsic components (e.g. C1‐C9), complement receptors (e.g. CR2 C3aR, C5aR), and complement regulatory proteins (e.g. CFH, CFI) (West and Kemper 2023). The complement system can be activated through classical, lectin, or alternative pathways, and all exert important roles in the regulation of tumor development (Afshar‐Kharghan 2017; Noris and Remuzzi 2013). As the central element of this system, C3 can be activated through all these three different pathways to release activated C3 (C3a) (Walport 2001a, 2001b). C3a is a pro‐inflammatory peptide, that triggers migration and activation of inflammatory cells by binding to the C3a receptor (C3aR) (Daffern et al. 1995; K. Li et al. 2008). The lectin cascade is activated through the pattern recognition molecule, mannose binding lectin, (MBL) that recognizes and binds to mannose residues in the fungal cell wall (Gracia et al. 2021). Aykut et al. have demonstrated that MBL activates C3 by binding to the glycans on the surface of *Malassezia sp*, thereby leading to the progression of pancreatic cancer (Aykut et al. 2019). However, this correlation between *Malassezia globosa* and MBL‐C3a‐C3aR in the context of breast cancer has not yet been elucidated.

Here, we began by studying the differences in levels of *Malassezia globosa* in patient breast cancer tissues wherein we found significantly higher levels in the tumor tissues vis a vis normal controls. Fungal levels had a positive outcome on tumor progression as revealed by fungal inhibitors in PDX models. *M. globosa* drove breast cancer tumor growth via activation of the MBL‐C3a‐C3aR pathway with both MBL ablation and C3aR inhibition reversing this effect. In toto, this study unravels a novel mechanistic role for the host mycobiome in regulating breast cancer progression and it opens up new avenues for therapy.

## 2 Materials and Methods

### 2.1 Clinical Samples

All cancerous tissues and matched noncancerous tissues were obtained from breast cancer patients at Jiangxi Cancer Hospital, which were collected from August 2021 to February 2023. A total of 20 patients (female) were included in this study with an average age of 45.6 years. Both normal tissues and tumor tissues were procured intraoperatively by board‐certified breast surgeons with extensive clinical experience, ensuring standardized and reliable tissue collection. Normal tissues were obtained from the non‐tumorous, peritumoral regions of the same patients undergoing mastectomy, located at least 2 cm away from the tumor margin, and confirmed histologically free of malignant cells. The diagnostic criteria complied with the 2018 edition of the Chinese Breast Cancer Diagnostic and Treatment Guidelines (Health Commission of PRC 2019). This investigation was conducted in accordance with the Declaration of Helsinki with written informed consent from all participants and also approved by the Ethics Committee of Jiangxi Cancer Hospital.

### 2.2 Quantitative Real‐Time Polymerase Chain Reaction (qRT‐PCR)

Tissues suspended in phosphate‐buffered saline (PBS) were vortexed and sonicated and then treated with Proteinase K (Thermo Fisher Scientific, Waltham, Massachusetts, USA). Total DNA was purified, and real‐time PCR was carried out using a SYBR Green PCR kit (Takara, Beijing, China). Primers were set as follows: ITS1F (5'‐CTTGGTCATTTAGAGGAAGTAA‐3'), ITS2 (5'‐GCTGCGTTCTTCATCGATGC‐3'); GAPDH (F: 5'‐TCAAGGCTGAGAACGGGAAG‐3'; R: 5'‐TGGACTCCACGACGTACTCA‐3'). Amplification procedures were 95°C for 5 min, 35 cycles (at 95°C for 45 s, at 60°C for 40 s, at 72°C for 30 s), and finally at 72°C for 5 min. Relative abundance of fungi was calculated via the comparative cycle threshold (CT) method (2^−ΔΔCt^) (Rao et al. 2013).

### 2.3 Animals and Tumor Models

BALB/c‐nude mice, wild type C57BL/6 and MBL‐null mice on a C57BL/6 background (female, 4–5 weeks old) were acquired from the HuBin Lab at the First Affiliated Hospital of Nanchang University. MBL‐C knockout mice were crossed with MBL‐A knockout mice to create MBL‐null mice. Animal experiments were executed based on guidelines of the Institutional Animal Care and Use Committee of Beijing Viewsolid Biotechnology Co. LTD and the NIH's Guide for the Care and Use of Laboratory Animals. All methods are reported in accordance with ARRIVE guidelines (https://arriveguidelines.org).

To generate the Patient‐Derived Xenograft (PDX) tumor model, BALB/c‐nude mice were subcutaneously inoculated with MDA‐MB‐231 cells (3 × 10^6^ per mouse) in the mammary fat pads, while wild‐type C57BL/6 and MBL‐null mice were similarly inoculated with E0771 cells (3 × 10^6^ per mouse). Four weeks after tumor implantation, mice were killed via cervical dislocation and the mammary tumors were removed for analysis.

### 2.4 Fungal Treatment

Mice received treatment with taxol (MedChemExpress, Shanghai, China) through intraperitoneal injection (1.2 mg twice/week). For ablating the mycobiome, mice were administered with 1 mg/mL Amphotericin‐B (MP Biomedicals, Beijing, China) via oral gavage daily for five consecutive days, followed by providing the mice with drinking water supplemented with 0.5 μg/mL Amphotericin‐B or without Amphotericin‐B (control group) for the duration of the experiment. For species‐specific repopulation experiments, *S. cerevisiae* (1 × 10^8^ CFU/mL; 7752, ATCC), *Candida sp*. (1 × 10^8^ CFU/mL; clinical isolate), *Malassezia globosa* (M. globosa; 1 × 10^8^ CFU/mL; MYA‐4612, ATCC), or *Aspergillus sp*. (1 × 10^8^ CFU/mL; clinical isolate) were utilized for mouse oral gavage after treatment of Amphotericin B. Recipient mice were implanted with the tumor cells 7 days after repopulation (J. Li et al. 2025), and control groups received oral gavage with PBS buffer (placebo) without M. globosa inoculation.

### 2.5 Oxidative Treatment of Malassezia Globosa

Mannose, a monosaccharide bearing vicinal hydroxyl groups, is selectively oxidized by sodium meta‐periodate (NaIO₄). The reagent cleaves the carbon–carbon bond between two adjacent hydroxyl‐bearing carbons (the vic‐diol moiety), converting each hydroxyl into an aldehyde while IO_4_^−^ is reduced to IO_3_^−^. This periodate oxidation severs the native mannopyranoside structure, thereby abolishing its recognition by mannose‐specific lectins and downstream binding events. M. globosa cells (1 × 10⁸ CFU) were harvested by centrifugation (3000 × *g*, 5 min, 4°C), washed twice with ice‐cold PBS, and resuspended in 1 mL PBS containing 10 mM sodium meta‐periodate (NaIO₄). The suspension was kept on ice in the dark for 30 min with gentle agitation to selectively oxidize vicinal mannose diols. Excess periodate was quenched by adding 100 µL of 10% (w/v) glycerol (5 min, ice). Cells were then pelleted, washed three times with PBS to remove reaction by‐products, and used immediately in downstream assays.

### 2.6 Immunohistochemistry (IHC) Staining

Formalin fixed samples were embedded, cut into sections, dewaxed and rehydrated. Next, antigen retrieval was executed with blocking for nonspecific antigens. After that, these sections were incubated with primary antibodies including anti‐Ki67 (1:1000, ab279653, Abcam), anti‐C3a (1:150, ab36385, Abcam) and anti‐C3aR (1:1000, ab317632, Abcam) at 4°C overnight. Next day, the secondary antibody (1:500, ab6823, Abcam) was appended to incubate for 30 min, followed by DAB addition and hematoxylin staining. A Leica DM 2500 microscope (Wetzlar, Germany) was utilized for observing the staining.

### 2.7 Cell Culture and Treatment

The human monocytic leukemia cell line (THP‐1) and breast cancer cell lines (MCF‐7, MDA‐MB‐231 cells and E0771 cells) and were bought from the Cell Bank of the Chinese Academy of Sciences (Shanghai, China). Dulbecco's Modified Eagle Medium (DMEM; Hyclone, Logan, UT, USA) containing 10% FBS (Gibco, Gaithersburg, MD, USA) and 1% penicillin‐streptomycin (Gibco) was used for culturing MDA‐MB‐231 and MCF‐7 cells. Roswell Park Memorial Institute 1640 Medium (RPMI1640; Hyclone) was used for culturing THP‐1 cells. All cell lines were placed at 37°C in a humidified incubator with 5% CO_2_. THP‐1 cells were treated with 200 nM PMA (Sigma‐Aldrich) for 24 h to induce them into M0 macrophages and the differentiated macrophages were named as M0 macrophages. C3aR antagonist (SB290157) was bought from ApexBio (Shanghai, China) and used at 2 μM for pretreating THP‐1 cells for 24 h.

### 2.8 Colony Formation Assay

MCF‐7 and MDA‐MB‐231 cells were seeded into six‐well plates. After 2 weeks of incubation, these cells were fixed with formaldehyde for 15 min and stained with 0.1% crystal violet for 30 min. Colonies were photographed with a digital camera and ImageJ software (National Institute of Health, Bethesda, MD, USA) was used to count the colonies.

### 2.9 Transwell Assay

Invasion and migration of MDA‐MB‐231 and MCF‐7 cells were evaluated with Transwell chambers (8 μm pore size; Corning, New York, NY, USA) coated with or without matrigel. Briefly, MDA‐MB‐231 and MCF‐7 cells in FBS‐free DMEM (200 μL) were seeded into the upper compartment of a 24‐well Transwell chamber and the lower compartment filled with DMEM medium (600 μL) containing 10% FBS. Following 24 h, 4% paraformaldehyde and 0.5% crystal violet were used to treat cells that adhered to the lower chambers. Stained cells were observed through an Olympus IX70 inverted microscope (Tokyo, Japan).

### 2.10 Western Blot

THP‐1 cells were lysed in RIPA buffer from Beyotime (Shanghai, China) and 20 μg of the total protein lysate was loaded and resolved in a 10% sodium dodecyl sulfonate‐polyacrylamide gel (Solarbio, Beijing, China) and transferred to PVDF membranes (Pall Corporation, New York, NYC, USA). Blocking was performed in 5% nonfat milk in PBST (PBS and 0.1% Tween‐20) and the membrane was incubated with the respective primary antibody overnight [anti‐CD206 (ab300621, Abcam), anti‐IL‐10 (ab133575, Abcam, Cambridge, MA, USA), anti‐ARG1 (ab133543, Abcam), and anti‐β‐actin (1:2000, ab8227, Abcam)]. Following washes and addition of the secondary antibody (1:5,000; SanYing, Beijing, China), blots were developed with a chemiluminescence reagent (Thermo Fisher Scientific), and quantified with the Image J software (NIH, USA).

### 2.11 Mannose‐Binding Detecting by Elisa

MaxiSorp 96‐well plates (Thermo Fisher) were coated overnight at 4°C with 100 µL per well of 5 µg mL⁻¹ mannose–BSA conjugate (Sigma–Aldrich, RAB0548) in 50 mM carbonate buffer pH 9.6. After three washes with PBST (PBS + 0.05% Tween‐20), wells were blocked with 200 µL 2% (w/v) BSA in PBST for 1 h at 37°C. Standard diluted recombinant protein or samples to be tested were added (100 µL per well) and incubated for 1 h at 37°C. Bound protein was detected with an HRP‐conjugated primary antibody (1:5000 in PBST, 100 µL, 1 h, 37°C) followed by 100 µL TMB substrate (15 min, RT). The reaction was stopped with 50 µL 2 M H₂SO₄, and absorbance was read at 450 nm (reference 630 nm) on a SpectraMax iD3 plate reader. All measurements were performed in triplicate; background absorbance from mannose‐free BSA‐coated wells was subtracted.

### 2.12 C3a Detecting by ELISA

C3a was measured with the Invitrogen Human C3a Platinum ELISA (Abcam, ab279352). Cell lysates (20 µg total protein per well) were assayed in duplicate on antibody‐coated plates. After 2 h at 37 degrees Celsius, wells were washed, incubated with biotinylated detection antibody (1 h) and HRP‐streptavidin (30 min), developed with TMB, stopped with 2 N H₂SO₄, and read at 450 nm. Concentrations were interpolated from a four‐parameter logistic standard curve and expressed as ng/mg protein (C3a).

### 2.13 Flow Cytometry

PMA‐induced THP‐1 cells were stained with anti‐CD206‐APC (San Diego, CA, USA) or anti‐CD163‐FITC (San Diego). Then a BD Accuri C6 flow cytometer was applied to analyze the number of CD206‐positive or CD163‐positive cells.

### 2.14 TIMER 2.0‐Based Correlation Analysis

Pan‐cancer mRNA expression values of the target gene (RNA‐seq TPM, tumor‐only samples) and corresponding QUANTISEQ‐derived M2‐macrophage infiltration scores were retrieved from the TIMER3.0 “Gene” module (https://timer.cistrome.org/). Samples lacking either parameter were excluded, leaving 10 897 tumors across 32 cancer types. Spearman rank‐correlation coefficients (ρ) and two‐tailed *p*‐values were computed with the built‐in TIMER algorithm; plots were exported directly from the web interface. A |ρ| ≥ 0.4 and *p* < 0.001 were pre‐specified as thresholds for biological relevance.

### 2.15 Statistical Analysis

GraphPad Prism 6 (GraphPad Software Inc., San Diego, USA) was utilized for conducting statistical analysis and data was exhibited as mean ± standard deviation (SD). Differential comparisons among multiple groups were carried out through a one‐way ANOVA, followed by Tukey's posttest. Difference comparisons between two groups were executed using the student's *t*‐test and a statistically significant difference was indicated as *p* < 0.05.

## 3 Results

### 3.1 Fungal Colonization Promotes the Progression of Breast Cancer In Vivo

**Figure 1:**
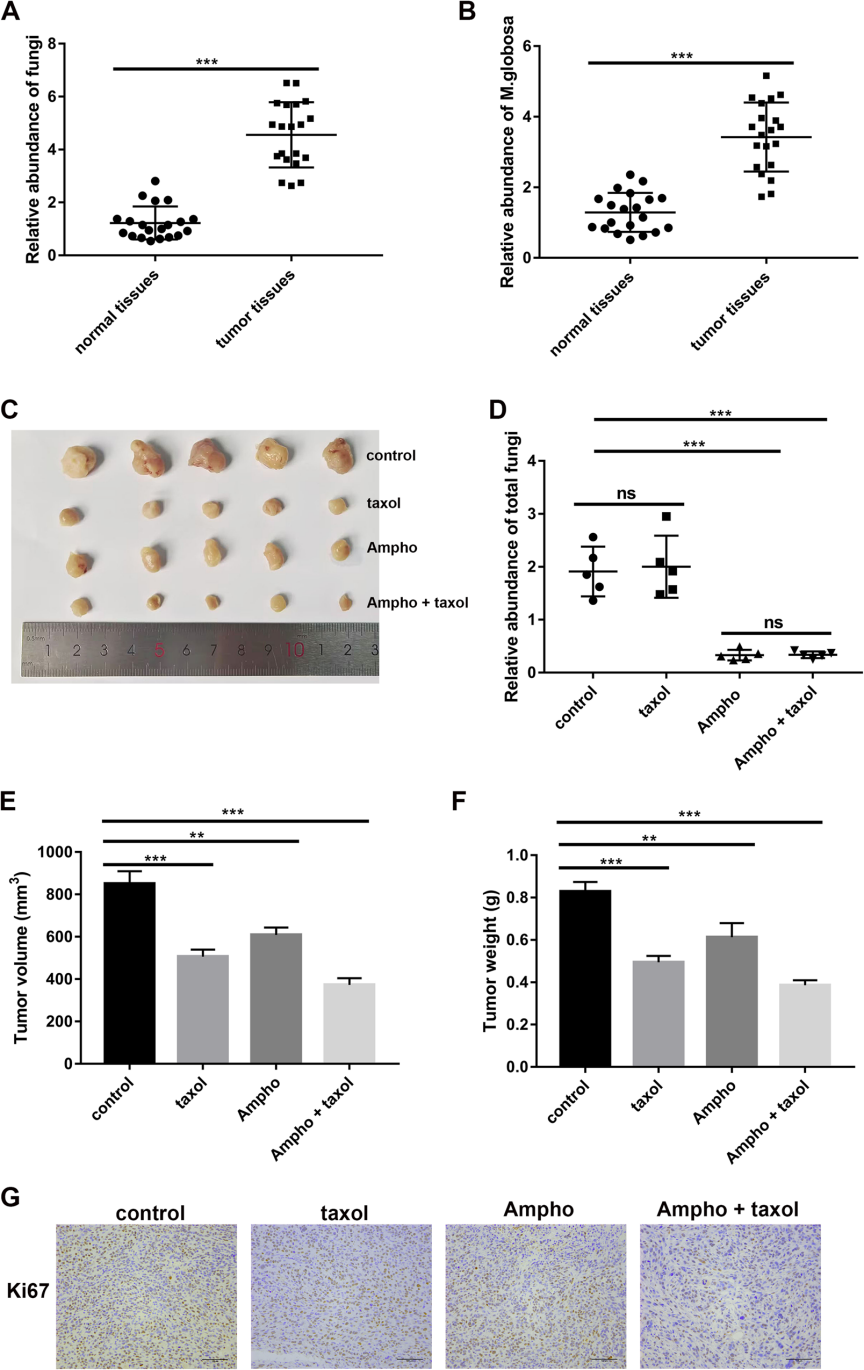
Fungal colonization facilitates breast cancer progression in vivo. (A, B) Relative abundance of fungi and Malassezia‐globosa was detected respectively by quantitative real‐time polymerase chain reaction (qRT‐PCR) in tumor tissues and normal tissues of breast cancer patients (*n* = 20 per group, triplicate times per sample). Two‐tailed paired *t*‐test. ***p < 0.001, **p < 0.01. (C–F) Female BALB/c nude mice bearing MDA‐MB‐231 cells were treated with taxol and Amphotericin B (Ampho), and tumor size, final volume, and weight were measured (*n* = 5 per group, triplicate times per sample). One‐way ANOVA test. ***p < 0.001, **p < 0.01. ns = not significant. (G) The protein expression of Ki67 in tumor tissues was detected using immunohistochemistry (*n* = 5, triplicate times per sample).

We began by studying *Malassezia globosa* levels in breast cancer patients and our qRT‐PCR results showed increased abundance of total fungi and *Malassezia globosa* in tumor tissues relative to normal tissues (*p* < 0.01, Supporting Information S1: Figure 1; Figure 1A,B). We then proceeded with attempting to decipher the relevance of increased fungal abundance in tumor tissues. For investigating the effect of fungi on breast cancer, the mycobiome was ablated through the fungal inhibitor (Amphotericin‐B) in and we discovered that tumor size, tumor volume (*p* < 0.01) and weight (*p* < 0.01) were markedly diminished by the administration of Amphotericin‐B or taxol (Figure 1C–F) in PDX mouse models. We also found an additive effect when taxol‐based chemotherapy was used alongside fungal ablation (*p* < 0.01, Figure 1C–F). Furthermore, the expression of Ki67 (a protein marker of cell proliferation) in tumor tissues of mice was significantly reduced in response to Amphotericin‐B and taxol (Figure 1G), thereby indicating the positive correlation between fungi abundance and tumor growth.

### 3.2 Malassezia Globosa Facilitates the Progression of Breast Cancer In Vitro

**Figure 2:**
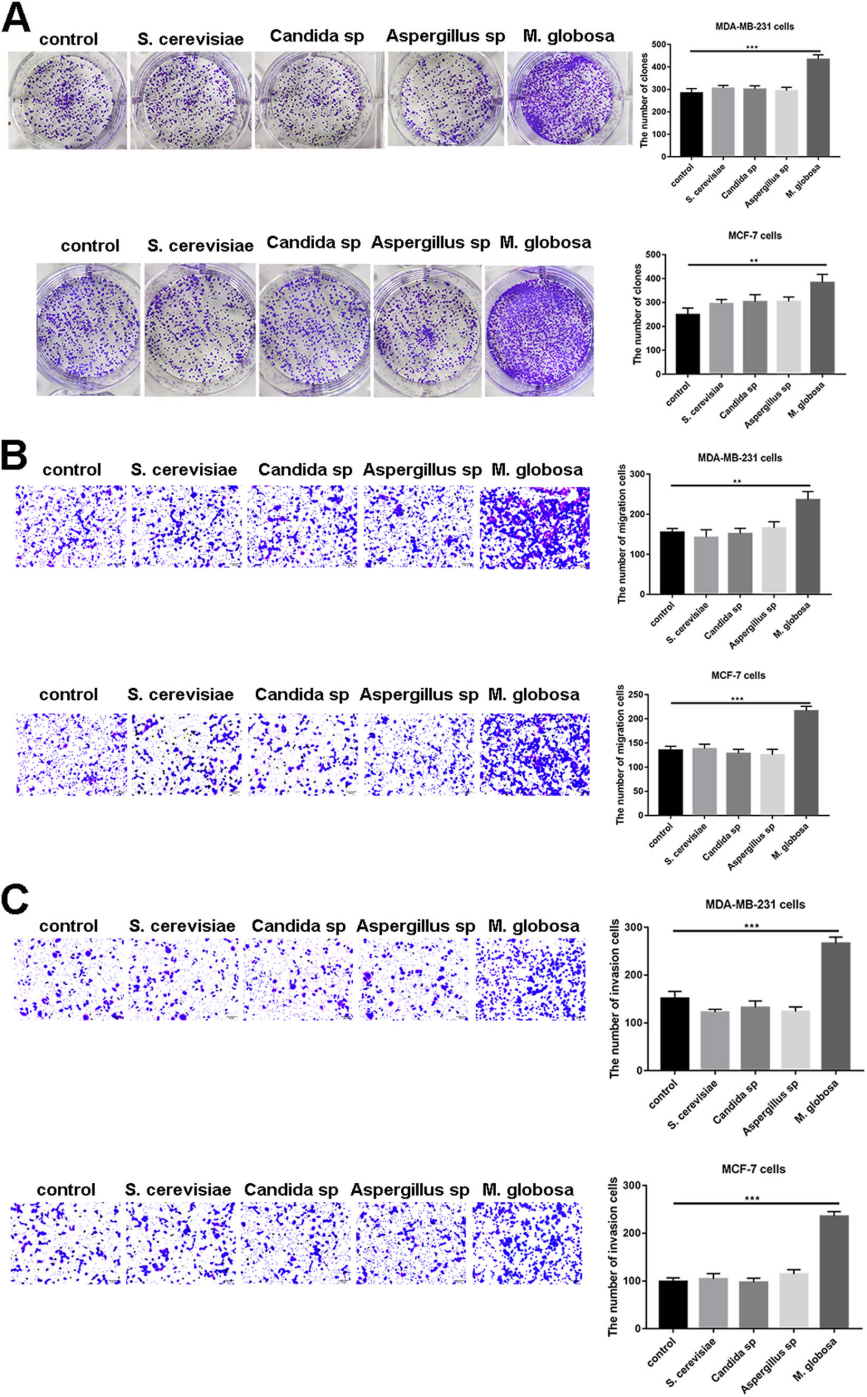
*Malassezia globosa (M. globosa)* promotes the progression of breast cancer in vitro. (A) Colony‐formation assay of MDA‐MB‐231 and MCF‐7 cells was performed after special treatments. (*n* = 5 per group, triplicate times per sample). One‐way ANOVA test. ***p < 0.001, **p < 0.01. (B, C) Transwell assay of MDA‐MB‐231 and MCF‐7 cells was performed to quantify cell invasion or migration after special treatments (*n* = 5 per group, triplicate times per sample). One‐way ANOVA test. ***p < 0.001, **p < 0.01.

Next, we moved onto exploring the influence of *Malassezia globosa* on breast cancer invasion and migration. For this, Amphotericin B‐treated breast cancer cells (MDA‐MB‐231 and MCF‐7 cells) were incubated with either *Malassezia globosa* or control fungi (*S. cerevisiae, Candida sp*, and *Aspergillus sp*) for 24 h, following which cell proliferation, migration and invasion were assessed. Colony formation assay displayed an increased number of colonies with *Malassezia globosa*, but not with *S. cerevisiae, Candida sp* or *Aspergillus sp* (*p* < 0.01; Figure 2A). *M. globosa* treated cells also showed enhanced invasion and migration in the Transwell assay, an observation that was not witnessed with *S. cerevisiae, Candida sp or Aspergillus sp* (*p* < 0.01; Figure 2B,C).

### 3.3 Malassezia Globosa Promotes Tumor Formation In Vivo

**Figure 3:**
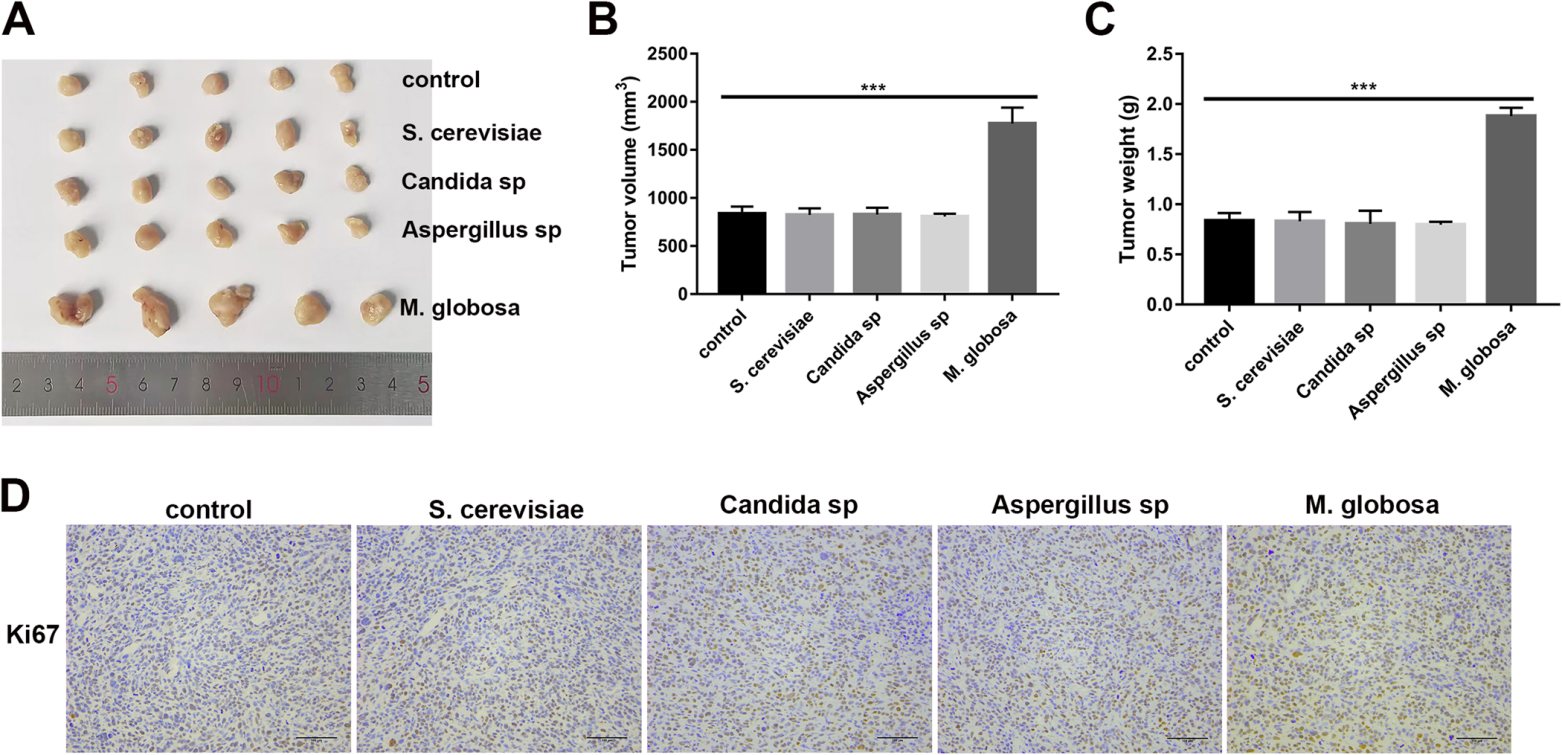
*Malassezia globosa (M. globosa)* contributes to tumor formation in vivo. (A–C) Female BALB/c nude mice bearing MDA‐MB‐231 cells were gavaged with 1 × 10^7^ CFU equivalents of *S. cerevisiae*, Candida sp., Malassezia globosa, or Aspergillus sp. following treatment with Amphotericin B, and tumor size, final volume, and weight were monitored (*n* = 5 per group, triplicate times per sample). One‐way ANOVA test. ***p < 0.001, **p < 0.01. ns = not significant. (D) The protein expression of Ki67 in tumor tissues of mice was detected using immunohistochemistry (*n* = 5 per group, triplicate times per sample).

Once we affirmed the positive role of *M. globosa* levels in promoting breast cancer growth, our subsequent effort was to procure further confirmatory data on its specific role in this process. To confirm that *Malassezia globosa* accelerated tumor progression in vivo, Amphotericin B‐treated mice were repopulated with *Malassezia globosa* or other control fungi (*Aspergillus sp, S. cerevisae*, or *Candida sp*.) and then implanted into the mammary fat pad of BALB/c mice as before. We found that *Malassezia globosa* exclusively accelerated tumor growth with repopulation by *Candida sp., S. cerevisae*, or *Aspergillus sp* showing no significant effect (Figure 3A–C). Similarly, consistent results were obtained when syngeneic tumor cells were implanted into wild‐type C57 mice (Supporting Information S1: Figure 2A–C). Likewise, only *Malassezia globosa* increased protein expression of the proliferative marker Ki67 in the tumor tissues of these mice (Figure 3D).

### 3.4 Malassezia Globosa Promotes the Progression of Breast Cancer Through the MBL‐C3‐C3a‐C3aR Signaling Pathway

**Figure 4:**
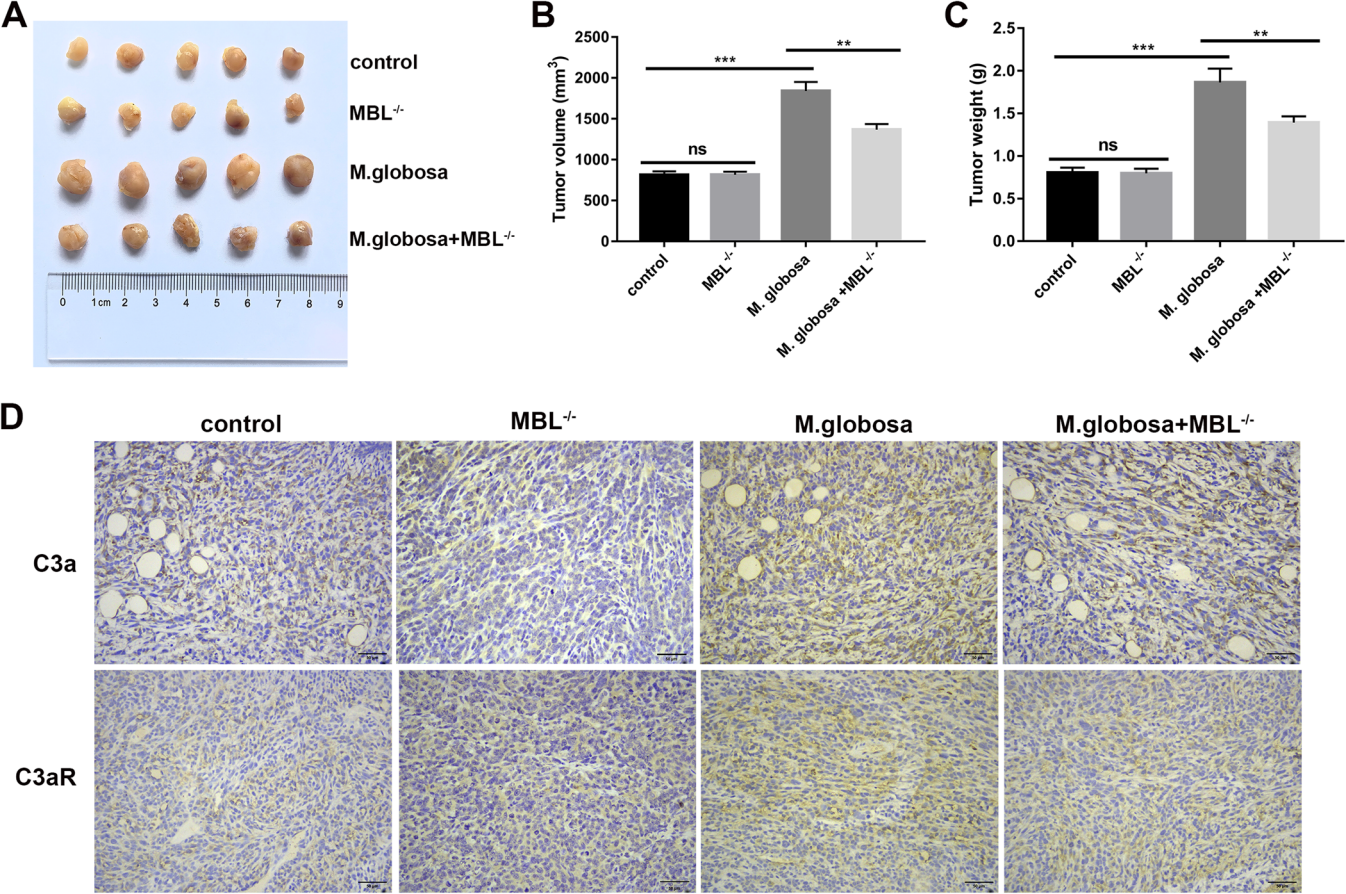
*Malassezia globosa* promotes the progression of breast cancer through the MBL‐C3‐C3a‐C3aR signaling pathway. (A–C) Tumor size, tumor volume, and tumor weight were observed in amphotericin B‐treated wild‐type C57 mice and MBL‐knockout mice inoculated with E0771 cells (*n* = 5 per group, triplicate times per sample). One‐way ANOVA test. ***p < 0.001, **p < 0.01, ns = not significant. (D) Protein expression of C3a and C3aR in tumor tissues of mouse model was detected using immunohistochemistry (*n* = 5 per group, triplicate times per sample).

A previous study showed that *M. globosa* contributes to pancreatic oncogenesis through activation of the pro‐inflammatory MBL‐complement pathogen recognition signaling pathway (Aykut et al. 2019). Thus, we then extrapolated the influence of *Malassezia globosa* on breast cancer cells in the context of MBL activation. To study this phenomenon, MBL‐knockout mice were implanted with breast cancer cells as before and tumor size, tumor volume and tumor weight were measured after administration of *Malassezia globosa*. Along anticipated lines, we found that the positive effect of *Malassezia globosa* on tumor growth was reversed with the ablation of MBL expression (Figure 4A–C). Subsequently, we then measured the expression of the downstream MBL signaling components, C3a and C3aR by IHC. We discovered that C3a and C3aR expression in tumor tissues of mice were increased after administration of *Malassezia globosa*, and were reversed by MBL knockout (Figure 4D). Collectively, these findings implied that *Malassezia globosa* facilitated tumor progression in breast cancer via regulation of the MBL‐C3‐C3a‐C3aR pathway.

### 3.5 Malassezia Globosa Induced‐M2 Macrophage Polarization Affects the Development of Breast Cancer In Vitro

**Figure 5:**
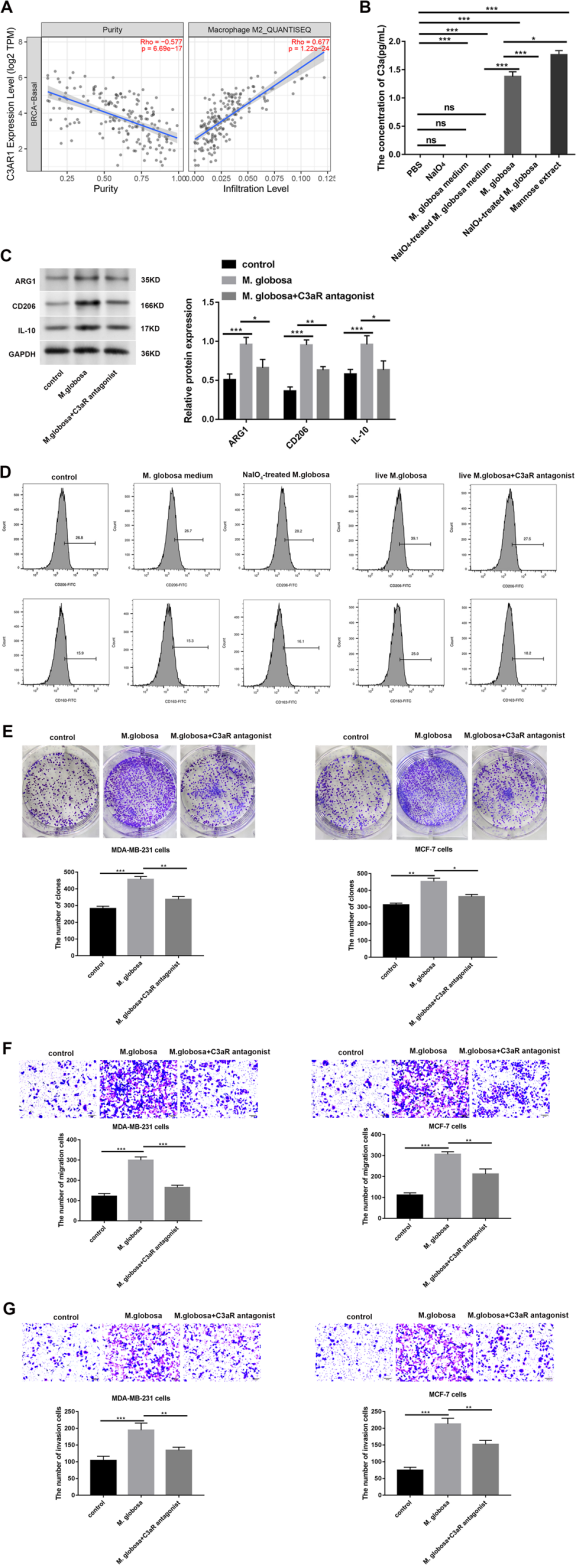
*Malassezia globosa* induced‐M2 macrophage polarization affects the development of breast cancer in vitro. (A) The correlation between C3AR1 and M2 macrophage infiltration was analyzed via the Timer database. (B) Secreted C3a levels in PMA‐induced THP‐1 cells after different treatments by Elisa detection (*n* = 5 per group, triplicate wells per sample). One‐way ANOVA test. ***p < 0.001, **p < 0.01. ns = not significant. (C) Western blot was used to examine protein expression of ARG1, CD206 and IL‐10 in PMA‐induced THP‐1 cells after specific treatments (*n* = 5 per group, triplicate times per sample). Two‐way ANOVA test. ***p < 0.001, *p < 0.05. (D) Flow cytometry was used to examine expression of CD206 and CD163 in PMA‐induced THP‐1 cells after specific treatments (*n* = 5 per group, triplicate times per sample). (E) Colony formation assay was used to assess proliferation of MDA‐MB‐231 and MCF‐7 cells (*n* = 5 per group, triplicate times per sample). One‐way ANOVA test. ***p < 0.001, **p < 0.01, and *p < 0.05. (F, G) Transwell assay was used to assess migration and invasion of MDA‐MB‐231 and MCF‐7 cells (*n* = 5 per group, triplicate times per sample). **p < 0.01, ***p < 0.001.

Through bioinformatic analysis, we discovered that C3AR1 expression (gene name for the C3aR protein) positively correlated with the infiltration of M2 macrophages (Figure 5A). M. globosa contain mannose, which can specifically bind to MBL (Supporting Information S1: Figure 2A) and promote macrophages to cleave C3 to generate C3a (Figure 5B). However, M. globosa treated with sodium periodate have their mannose components completely destroyed, thus failing to bind to MBL and inhibiting the cleavage of C3 to generate C3a (Supporting Information S1: Figure 2A; Figure 5B). We then investigated whether *M. globosa* affects macrophage polarization via the MBL‐C3‐C3a‐C3aR pathway. Data from the western blots indicated that protein levels of the M2 macrophage marker proteins (ARG1, CD206 and IL‐10) were significantly elevated in PMA‐induced THP‐1 cells after treatment with *Malassezia globosa* and were reversed by the addition of a C3aR antagonist (*p* < 0.05, Figure 5C). Flow cytometry showed that protein levels of the M2 macrophage markers (CD206 and CD163) were significantly upregulated in PMA‐induced THP‐1 cells after treatment with *Malassezia globosa*, which were reversed by treatment with the C3aR antagonist or sodium periodate (Figure 5D). Furthermore, after treating tumor cells with the culture medium conditioned with *Malassezia globosa*‐treated THP‐1 cells for 24 h, proliferation, migration and invasion of MDA‐MB‐231 and MCF‐7 cells were all elevated and again reversed in the presence of the C3aR antagonist (*p* < 0.05, Figure 5E–G), thus reaffirming the activation of the MBL‐complement pathway by *M. globosa*.

## 4 Discussion

A growing body of evidence suggests that colonization in the gut microbiota is an important factor in breast cancer occurrence and development (Nandi et al. 2023; Plaza‐Díaz et al. 2019; Ruo et al. 2021). Fungi, in particular, have been associated with colitis (Iliev et al. 2012), asthma (Wheeler et al. 2016) and colon cancer (Malik et al. 2018) and prior reports have unveiled carcinogenic roles for fungi. For instance, fungi have been shown to accelerate development of pancreatic cancer (Dambuza and Brown 2019) and *A. fumigatus* infection promotes tumor growth in an ectopic mouse model of breast cancer (Sohrabi et al. 2010). The fungus, *Malassezia* is a known component of the microbiome in breast cancer and (Saftien et al. 2023) intra‐tumoral *Malassezia globosa* has been associated with a shorter overall survival of breast cancer patients (Narunsky‐Haziza et al. 2022). Despite this correlative observation, a robust understanding on the mechanistic basis by which *Malassezia* regulates breast cancer progression is yet nebulous. In the present study, we attempted to decipher the mechanistic link between the fungus *Malassezia globosa* and breast cancer progression. Through qRT‐PCR in tumor tissues from breast cancer patients, we discovered an increased abundance of *M. globosa* in cancer tissues as opposed to normal tissues, which may be associated with breast cancer occurrence. The presence of *M. globosa* in these tumor samples correlated with a positive outcome on tumor growth as Amphotericin‐B was able to significantly reduce tumor growth, volume, weight and Ki67 expression in PDX mouse models.

We then investigated the mechanisms by which *Malassezia globosa* promotes the progression of breast cancer. Prior evidence has uncovered the coordinated involvement of the innate immune receptors in recognizing *Malassezia globosa* and orchestrating phagocyte antimicrobial responses (Wolf et al. 2021). The C3a‐C3aR innate immune signaling component has been shown to exert a positive role in melanoma, ovarian cancer, and lung cancer (Cho et al. 2014; Nabizadeh et al. 2016). More importantly, C3a‐C3aR signaling accelerated lung metastasis formation from breast cancer (Markiewski et al. 2008; Shu et al. 2020). In view of these findings, we hypothesized that *Malassezia globosa* promoted the progression of breast cancer via activation of MBL‐C3‐C3a‐C3aR signaling. Here, we observed that the positive effects of *Malassezia globosa* on tumor growth, C3a and C3aR expression were reversed in MBL knockout mice, suggesting that *Malassezia globosa* facilitated breast cancer progression through the MBL‐C3‐C3a‐C3aR signaling cascade. This observation is similar to the notion that the *Malassezia* activates the MBL‐complement signaling network to facilitate pancreatic cancer progression (Aykut et al. 2019).

Macrophages are indispensable innate immune cells that can polarize into an M1‐ or M2‐ phenotype (Wynn and Vannella 2016). Tumor‐associated macrophages exhibit mainly a M2‐like phenotype and facilitate tumor progression (Pathria et al. 2019; Schreiber et al. 2011). A previous study has revealed that gut mycobiome colonization promotes M2 macrophage polarization via fungi‐induced PGE2 (Kim et al. 2014). In this study, we discovered that *Malassezia globosa* induced polarization of M2 macrophages. At the same time, we observed a significant positive correlation between M2 macrophage infiltration and C3AR1 expression. The invasive potential of breast cancer cells was enhanced in culture medium from *Malassezia globosa*‐treated THP‐1 cells, which was rescued by a C3aR antagonist. Taken together, we infered that *Malassezia globosa* may drive breast cancer progression through M2 polarization of the macrophages via activation of the MBL‐C3‐C3a‐C3aR signaling pathway. To the best of our knowledge, this is the first such study to clarify the specific mechanistic role of *Malassezia globosa* in breast cancer. Targeted reduction of *Malassezia* may work to delay tumor progression and metastasis in patients suffering from breast cancer.

Given the toxicity associated with Amphotericin B, this study is limited to proof‐of‐concept evidence, and future work should prioritize safer anti‐fungal or immunomodulatory approaches. Moreover, this study can only suggest the potential association between fungal burden and breast cancer correlational nature of our clinical data limited by the lack of evidence from large‐scale patient cohort studies.

## 5 Conclusions

In summary, fungi potentiate tumorigenesis, and we confirmed an association between *Malassezia globosa*, and M2 macrophage polarization in breast cancer. Moreover, this study offers evidence that Malassezia promotes tumor progression through the pro‐inflammatory MBL‐C3a‐C3aR signaling pathway thereby providing new insights into the relationship between fungi and breast cancer.

## Author Contributions

Conceptualization: Chongwu He, Jing Chen, Jun Zou, Tenghua Yu. Data curation: Ruibo Tian, Xiaoqiang Zeng, Qinyuan Han, Changan Jiang. Formal Analysis: Ruibo Tian, Xiaoqiang Zeng, Qinyuan Han, Changan Jiang. Funding acquisition: Tenghua Yu. Writing – original draft: Chongwu He, Jing Chen. Writing – review and editing: all authors.

## Funding

This work was Supported by the Research Open Fund Project of Jiangxi Cancer Hospital (Grant Number: KFJJ2023ZD01, Grant Number: KFJJ2023YB06); the General Program of the Natural Science Foundation of Jiangxi Province (20252BAC240417); the Science and Technology Research Project of Jiangxi Provincial Department of Education (Grant Number: GJJ2208202); the Jiangxi Province Gan Po Talent Support Program (Grant Number: 20232BCJ23035); the Jiangxi Cancer Hospital Doctoral Start‐up Fund (Grant Number: BSQDJ202309).

## Ethics Statement

This investigation was conducted in accordance with the Declaration of Helsinki with written informed consent from all participants and also approved by the Ethics Committee of Jiangxi Cancer Hospital. Animal experiments were executed based on guidelines of the Institutional Animal Care and Use Committee of Beijing Viewsolid Biotechnology Co. LTD and the NIH's Guide for the Care and Use of Laboratory Animals.

## Conflicts of Interest

The authors declare no conflicts of interest.

## Supporting information


**Supporting Figure 1:** ITS‐PCR was performed on tumor tissues and normal tissues of breast cancer patients (*n* = 20 per group, triplicate times per sample), and fungal load was expressed as 2^–ΔCt relative to human β‐actin. Two‐tailed paired *t*‐test. ***P < 0.001, **P < 0.01.

**Supporting Figure 2:** (A–C) Female C57BL/6 WT mice bearing E0771 tumors were gavaged with 1 × 10⁷ CFU equivalents of *S. cerevisiae*, Candida sp., Malassezia globosa, or Aspergillus sp. following treatment with Amphotericin B, and tumor size, final volume, and weight were monitored (*n* = 5 per group, triplicate times per sample). One‐way ANOVA test, ***P < 0.001, **P < 0.01. ns = not significant.

## Data Availability

The data that support the findings of this study are available from the corresponding author upon reasonable request.
