# Supplementary figures and images for: Fungal Colonization by Malassezia globosa Promotes Breast Cancer Progression and M2 Macrophage Polarization Through the MBL‐C3a–C3aR Signaling Pathway
Source: Microbiologyopen. 2025 Dec 11;14(6):e70193. doi: 10.1002/mbo3.70193 (PMC12698337; doi:10.1002/mbo3.70193)

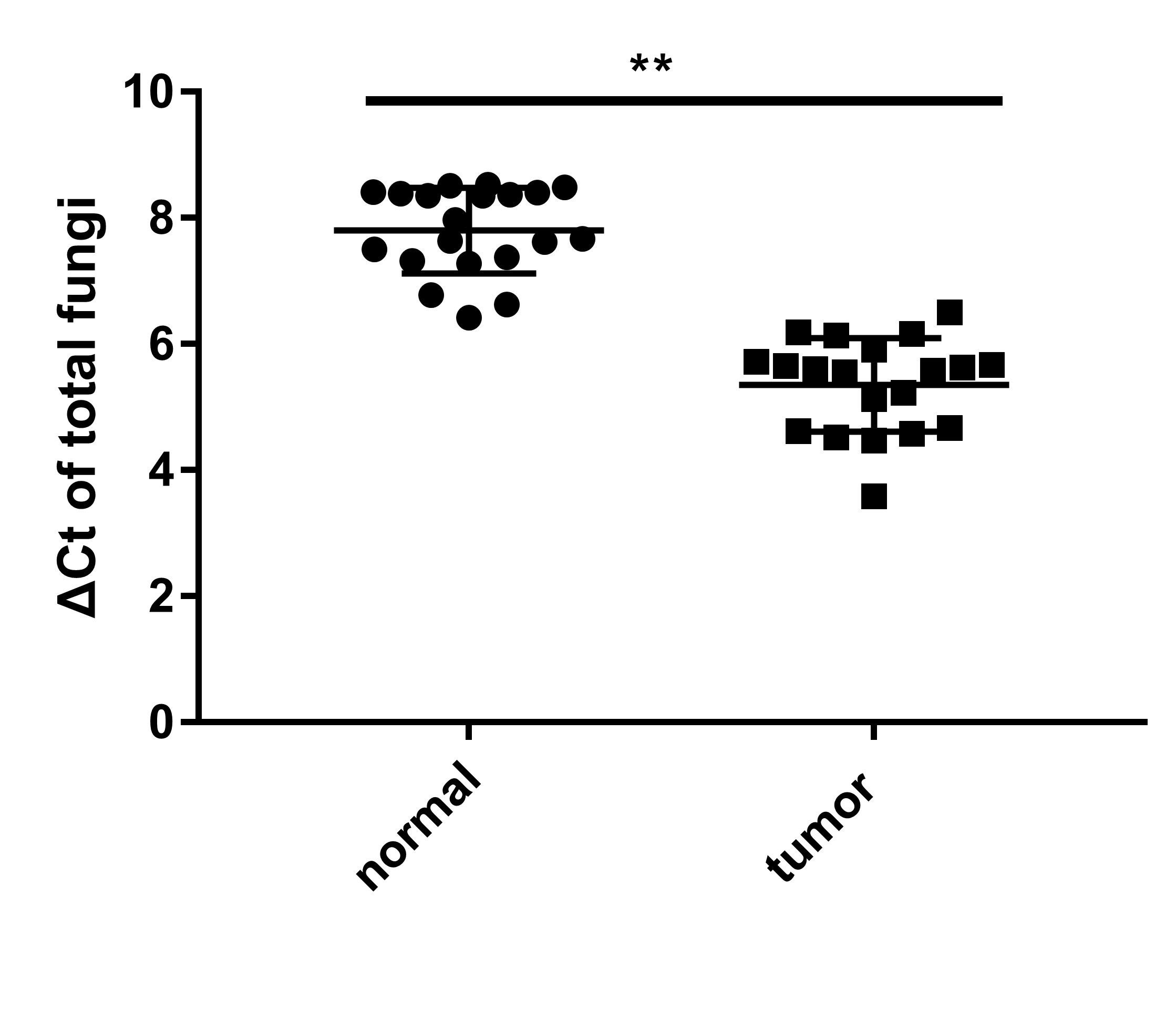

Supplement: Supplementary file 1 — Supporting Figure 1: ITS‐PCR was performed on tumor tissues and normal tissues of breast cancer patients (n = 20 per group, triplicate times per sample), and fungal load was expressed as 2^–ΔCt relative to human β‐actin. Two‐tailed paired t‐test. ***P < 0.001, **P < 0.01. [file MBO3-14-e70193-s002.tif]

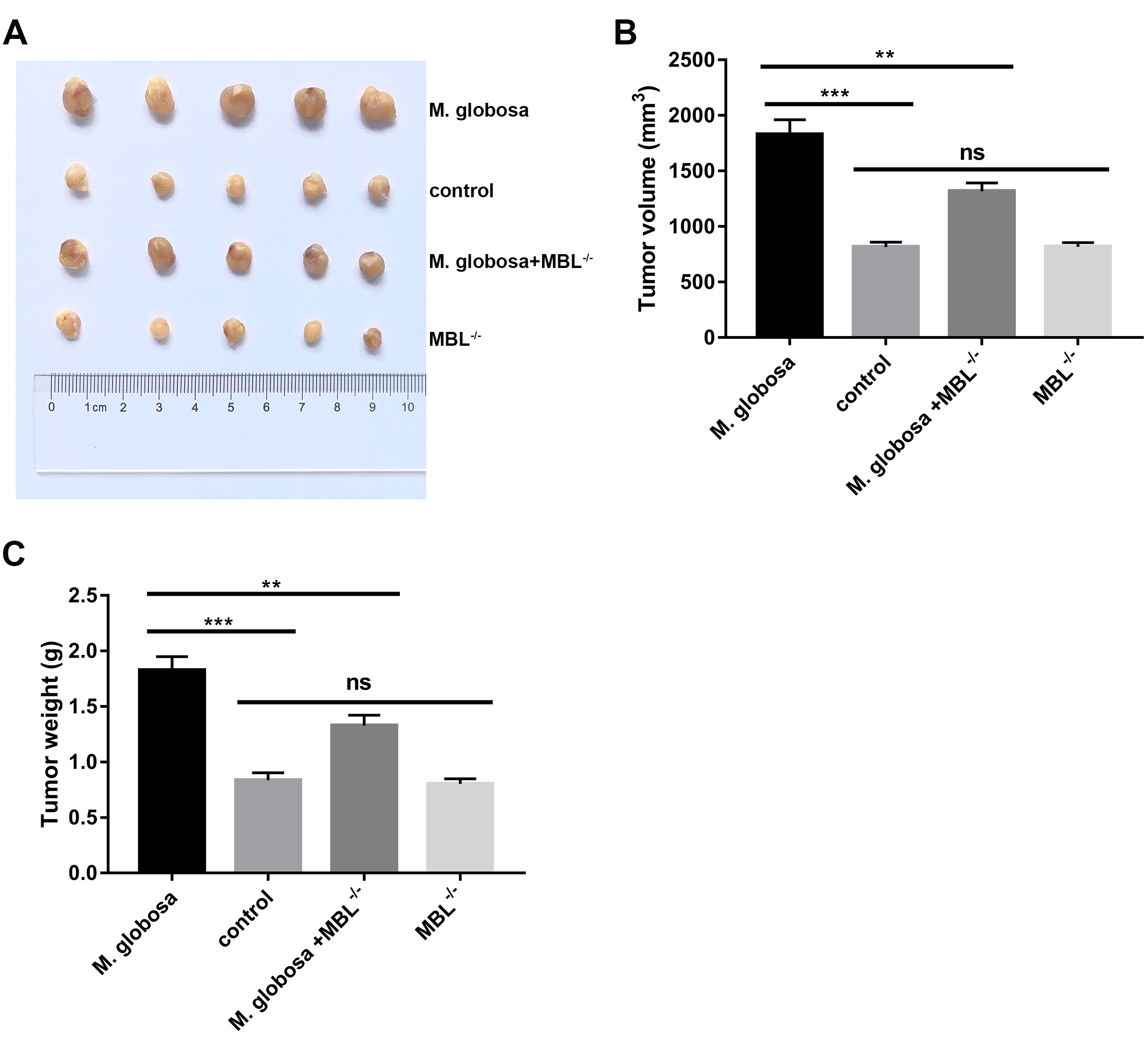

Supplement: Supplementary file 2 — Supporting Figure 2: (A–C) Female C57BL/6 WT mice bearing E0771 tumors were gavaged with 1 × 10⁷ CFU equivalents of S. cerevisiae, Candida sp., Malassezia globosa, or Aspergillus sp. following treatment with Amphotericin B, and tumor size, final volume, and weight were monitored (n = 5 per group, triplicate times per sample). One‐way ANOVA test, ***P < 0.001, **P < 0.01. ns = not significant. [file MBO3-14-e70193-s001.tif]
